# Electronic Structure and Vibrational Properties of Indenotetracene‐Based Crystal

**DOI:** 10.1002/jcc.70141

**Published:** 2025-05-24

**Authors:** Federico Coppola, Raoul Carfora, Nadia Rega

**Affiliations:** ^1^ Scuola Superiore Meridionale Napoli Italy; ^2^ Department of Chemical Sciences University of Napoli Federico II Napoli Italy; ^3^ Istituto Nazionale Di Fisica Nucleare, sezione di Napoli Napoli Italy

**Keywords:** DFT and TD‐DFT calculations, diarylindenotetracene crystal, electronic and vibrational properties, intermolecular interactions, organic semiconductors

## Abstract

Asymmetrically substituted indenotetracene crystals are promising nonfullerene electron transport materials for organic photovoltaics, offering potential improvements in efficiency and stability. In this work, we present a first‐principle investigation of the electronic and vibrational properties of a diarylindenotetracene system functionalized with two methoxy groups (hereafter *DimethoxyASI*). Single‐crystal X‐ray diffraction analysis [reported in *J. Org. Chem. 2018, 83, 4, 1828*] reveals a monoclinic ${\text{P2}}_{1}/\text{c}$ structure with an interplanar distance of 3.76 Å, providing insight into the molecular packing and intermolecular interactions that govern the solid‐state organization. Notably, for the first time, in this work we identify two distinct dimeric species within the crystalline lattice by a structural and electronic analysis, each exhibiting different intermolecular arrangements that significantly influence both the electronic structure and vibrational properties of the material. Density functional theory (DFT) and time‐dependent density functional theory (TDDFT) calculations provide insight into the molecular packing, electronic states, and vibrational characteristics of the crystal. The theoretical absorption spectrum, obtained from TDDFT calculations, features three main electronic transitions centered at 530, 360, and 275 nm, displaying a mixed character of localized excitations and charge‐transfer contributions. The vibrational properties, investigated through phonon density of states calculations at the DFT level, highlight well‐defined spectral features. While most vibrational modes remain consistent between monomeric and dimeric configurations, significant deviations emerge in the low‐frequency region, where intermolecular interactions and crystal packing effects play a crucial role. Furthermore, the two dimeric species exhibit distinct electronic properties beyond their geometric differences. A key distinguishing factor is the transition electric dipole moments (TEDMs), which governs the probability and polarization of electronic transitions. Our analysis reveals that the TEDMs magnitude and orientation vary significantly between the two dimeric species, suggesting that they may interact differently with polarized light. These differences provide new insight into the role of molecular aggregation in shaping the optical response of organic semiconductors and highlight the impact of polymorphism on their electronic properties. Overall, this study underscores the intricate relationship between molecular packing, electronic structure, and vibrational properties in indenotetracene‐based materials, contributing to a deeper understanding of their potential applications in optoelectronic devices.

## Graphical abstract

This study explores the electronic, vibrational, and optical properties of dimethoxy‐substituted indenotetracene crystals using DFT and TDDFT. We identify two distinct dimeric structures, highlighting their impact on molecular packing, transition dipole moments, and electronic states, providing new insights into the role of polymorphism in organic semiconductors.

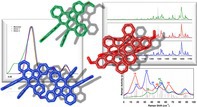

## 1 Introduction

**FIGURE 1:**
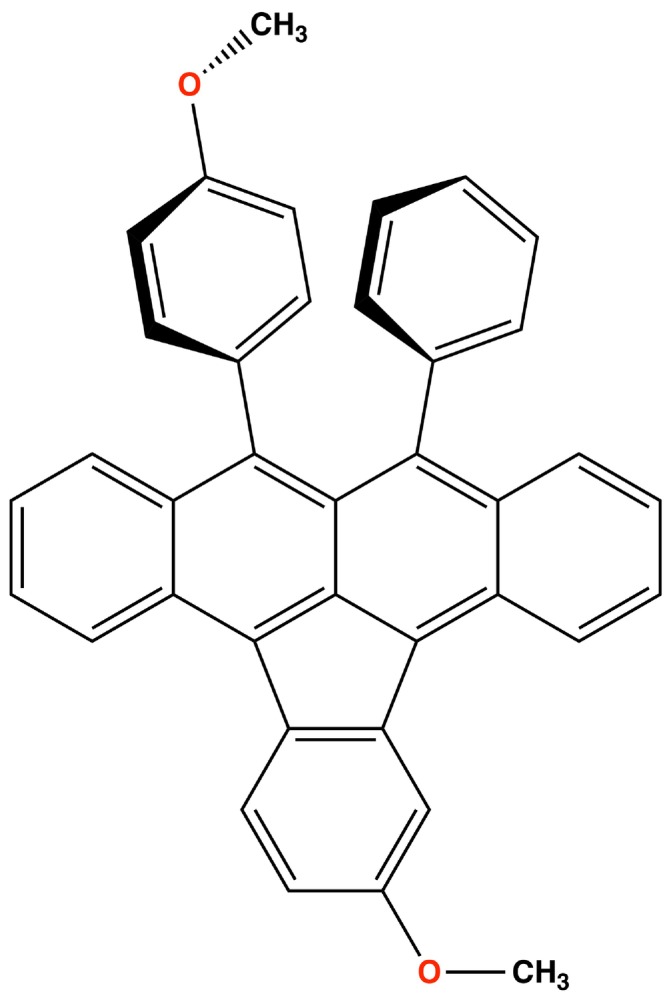
Monomeric structure of the electron‐deficient asymmetrically substituted diarylindenotetracene, IUPAC name: *2‐Methoxy‐9‐(4‐methoxyphenyl)‐10‐phenylindeno[1,2,3‐fg]‐tetracene*, named *DimethoxyASI*.

The urgent global demand for clean and sustainable energy sources to mitigate the impact of climate change highlights the need for innovative solutions. Among the various emerging technologies, organic electronics has attracted considerable attention for its potential to efficiently convert solar energy into usable forms [1, 2, 3, 4, 5]. This field offers promising pathways to renewable, affordable, and environmentally friendly energy sources that can be now safely integrated into modern applications and devices [6, 7, 8, 9, 10, 11]. Despite the abundance of solar energy, its utilization remains inadequate to meet the growing global energy demand, necessitating the development of advanced organic photovoltaic (OPV) materials [12, 13, 14]. A crucial aspect of this effort is the rational design of novel electron donor (*p*‐type) and electron acceptor (*n*‐type) molecules. Historically, perylene diimides and congeners [11, 15, 16, 17, 18, 19] and fullerene ${\text{C}}_{60}$ [20, 21, 22] derivatives, for example, have dominated OPV research due to their high electron affinity and excellent solid‐state properties, including isotropic charge transport [23, 24, 25, 26, 27, 28, 29, 30, 31]. However, these materials have inherent limitations. In particular, fullerene‐based acceptors suffer from poor stability, high production costs, and weak absorption in the visible spectrum. These drawbacks have prompted the search for new nonfullerene acceptors with better tunability and superior performance for next‐generation OPVs [32, 33, 34, 35, 36, 37, 38, 39, 40]. Organic electronics, beyond photovoltaics, offer several advantages over traditional inorganic materials, including flexibility, lightweight fabrication, cost‐effectiveness, and tunable optoelectronic properties. The ability to functionalize molecular cores with tailored substituents allows for precise control over the frontier molecular orbital gap (HOMO–LUMO), crystalline structure, light absorption characteristics, solubility, photostability, and charge transport efficiency [41, 42, 43, 44, 45]. Achieving optimal performance in OPV materials requires a deep understanding of energy transfer and charge transport mechanisms. In this regard, the synergistic combination of computational modeling [46, 47, 48, 49], and time‐resolved vibrational spectroscopy [50, 51, 52], provides a powerful framework for accurately predicting and elucidating structure–function relationships, offering deeper insights into the underlying electronic and dynamical properties. This approach enables a fundamental understanding of the interplay between nuclear and electronic properties, laying the foundation for future investigations into the dynamical aspects of photoinduced processes. Recent studies have identified indene‐based compounds as promising alternatives to fullerene acceptors, demonstrating their potential in various photovoltaic applications [53, 54]. In this context, we investigate the electronic and vibrational properties of a recently synthesized diarylindenotetracene derivative, asymmetrically functionalized with two methoxy groups, through first‐principle calculations. The molecular structure, which includes a tetracene backbone fused with a methoxy‐substituted indene and phenyl rings, favors electron acceptance due to its anti‐aromatic 4n π‐system. See Figure 1. Previous theoretical research [55] by some of the authors has provided a comprehensive analysis of photo‐induced charge‐transfer (CT) states and their ultrafast dynamics in asymmetrically substituted indenotetracene molecules through real‐time time‐dependent density functional theory (RT‐TDDFT) simulations [56, 57, 58, 59, 60]. The study revealed how different electron‐donating substituents (methyl or methoxy groups) can fine‐tune electronic energy levels and influence charge recombination pathways, which is crucial for optimizing organic photovoltaic materials.

The model system investigated in this work is an asymmetrically substituted diarylindenotetracene derivative bearing two methoxy groups, belonging to the class of alternant hydrocarbons, promising as *n*‐type compounds which can exploit photoinduced CT states to potentially undergo also singlet fission (SF) as well [61, 62, 63]. SF, a process wherein a singlet exciton is converted into two triplet excitons, requires multichromophoric systems with specific energy level alignments, although it can also occur intramolecularly [64, 65, 66, 67, 68, 69]. This mechanism has significant potential to enhance solar cell efficiency by effectively doubling the number of excitons generated per absorbed photon [70, 71, 72, 73]. In particular, SF can help overcome the Shockley‐Queisser limit [74], which defines the maximum theoretical efficiency of a single‐junction solar cell due to thermodynamic constraints on energy conversion. By generating multiple charge carriers from a single high‐energy photon, SF reduces thermalization losses, providing a viable strategy to improve photovoltaic performance beyond conventional limits.

We investigate the electronic and vibrational properties with DFT and TD‐DFT ab initio calculations. A detailed analysis of the crystal structure reveals the presence of two distinct dimeric arrangements: one in which the indenotetracene backbones exhibit significant overlap, and another where they are more separated, but with closer molecular planes. In this context, our investigation aims to elucidate the structural and electronic differences between these dimers and contribute to the characterization of their polymorphic mixture. Particular attention is given to the low‐energy absorption band in the UV–Vis spectrum, where TD‐DFT calculations indicate that the nature and transition probability of electronic transitions differ between the two dimers, altering the relative contributions of local excitations and (inter‐ and/or intra‐) charge‐transfer states. Notably, the calculation of transition electric dipole moments for both dimers has provided key insight into their selective recognition and excitation, particularly in the presence of single‐crystal polymorphism. Our results suggest that, for a fixed crystal orientation, selective excitation of one dimer over the other is theoretically achievable. Beyond singlet‐state properties, we also explored the lowest energy triplet states to assess the potential for singlet fission. In particular, we verify whether the energetic criterion for SF, $\text{E}({\text{S}}_{1})\geq 2\text{E}({\text{T}}_{1})$, is met. Our calculations reveal that while standard linear response TD‐DFT calculations underestimates triplet energies, alternative approaches such as ΔSCF and the Tamm‐Dancoff approximation provide more reliable results. These findings emphasize the importance of an accurate theoretical description of triplet state in evaluating the SF potential in OPV materials. Furthermore, we characterize in detail the Raman spectrum identifying and assigning the main Raman features in the 800–$1600\;{\text{cm}}^{-1}$ range through DFT Raman activity calculations. Special focus is placed on the low‐frequency spectral region (5–$150\;{\text{cm}}^{-1}$), THz Raman spectroscopy (0.2–5 THz) for short [75, 76, 77], which is particularly sensitive to intermolecular vibrations. Given that lattice phonon Raman spectroscopy can distinguish polymorphs based on their unique intermolecular vibrational modes [78, 79, 80], our study highlights the influence of the distinct local environments experienced by the two dimers. This work not only advances the understanding of the structural and electronic properties of diarylindenotetracene derivatives but also underscores the role of vibrational spectroscopy in probing polymorphism at the molecular level.

## 2 Methods and Computational Details

The structural and optical properties of the *DimethoxyASI* monomer and its higher homologues were characterized using density functional theory (DFT) [81, 82, 83] and its time‐dependent extension in the linear response formalism (LR‐TDDFT) [84, 85, 86, 87, 88, 89, 90, 91, 92], selecting a computational approach that ensures a reliable description while maintaining a balanced trade‐off between accuracy and computational cost. Ground state energies, gradients and higher order energy derivatives (infrared intensities and Raman activities) were computed for both the monomer and dimer in gas phase, the crystallographic structures of *DimethoxyASI* species were retrieved from Purvis et al. [54]. Calculations were performed using the hybrid Becke, 3‐parameter, Lee‐Yang‐Parr (B3LYP) [93] density functional with a double‐zeta basis set with diffuse functions on heavy atoms and d and p polarization functions on all atoms (6‐31+G(d,p)) [94, 95]. The crystallographic structure was refined through constrained geometric optimizations. Two iterative optimization procedures were adopted: in the first, all atomic coordinates were fixed except for those of the indenotetracene core, while in the second, only the two peripheral phenyl rings were allowed to relax. This approach allowed a better relaxation of C=C, C‐O, and C‐H bond lengths while preserving the relative orientation and molecular plane separation of the monomer and dimer structures, as observed in the crystallographic lattice. The convergence criteria adopted in this study are reported in Table S1 in ESI. Subsequently, TDDFT calculations, within the linear response formalism, were performed using the range‐separated hybrid (RSH) generalized gradient approximation (GGA) CAM‐B3LYP functional (19% exact Hartree‐Fock exchange increasing with ${\text{r}}_{12}$ to 65%) [96, 97, 98, 99, 100, 101] to characterize the energetic landscape (first 40 singlet states) and to better describe any charge transfer nature in low‐lying excited states of both spin multiplicities. The excited electronic states of extensive delocalized and multicenter chromophoric systems are characterized by multiple pairs of canonical molecular orbitals (MOs) with comparable CI coefficients. To facilitate their interpretation, natural transition orbitals (NTOs) [102] were computed to provide a more intuitive description of electronic redistribution in terms of reduced number of *hole‐electron* pairs. For all cases, the stability of single determinant for the 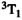 state was assessed [103, 104] and energies were also computed as the differences in the self‐consistent field energies (ΔSCF) between the singlet (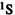) and triplet (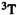) spin states. TDDFT calculations were further carried out using the Tamm–Dancoff approximation [105, 106, 107, 108, 109, 110] (TDDFT/TDA) at the CAM‐B3LYP/6‐31+G(d,p) level solving for both singlet and triplet excitations. The cartesian coordinates of the monomeric and dimeric equilibrium geometries are reported in Table S4–S6 in ESI. All presented calculations were performed using the Gaussian electronic structure software package [111].

## 3 Results and Discussion

### 3.1 Crystallographic Analysis and Molecular Packing

**FIGURE 2:**
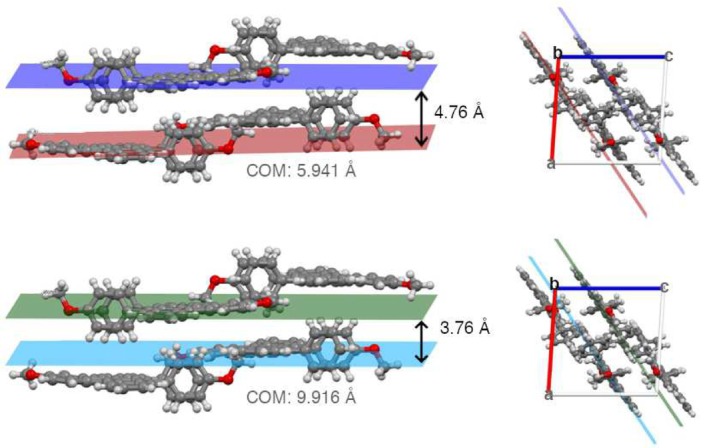
Two different dimeric arrangements identified in the crystallographic structure [54]. Top left: structure called *Dimer 1* in custom view to enhance the molecular planes and along the b‐axis (top right). Bottom left: structure called *Dimer 2* in custom view to enhance the molecular planes and along the b‐axis (bottom right). The center of mass distance (COM) between the two subunits that identify the molecular planes is reported in gray for each type.

The analysis of X‐ray diffraction experiments performed by Douglas and coworkers [54] showed that the crystal structure of *DimethoxyASI* belongs to the monoclinic ${\text{P2}}_{1}/\text{c}$ space group, with a measured π‐spacing of 3.76 Å. In this work we carefully analyzed the crystallographic structure of *DimethoxyASI*and identified at least two distinct dimeric arrangements. To characterize these dimers, we considered both the interplanar distance between the molecular planes passing through the indenotetracene core and the center‐of‐mass distance between the two monomers. These metrics provide a clear distinction between the two dimers, highlighting differences in molecular stacking and relative displacement. The first dimer (see Figure 2, top panel) exhibits partial overlap between the peripheral rings of one monomer and the backbone of the other, with a π‐π distance is 4.76 Å. The second dimeric structure (see Figure 2, bottom panel) where the monomers are more laterally displaced, while their molecular planes are closer by approximately 1.00 Å.

### 3.2 Electronic Landscape in the UV–Vis Absorption Spectrum

**FIGURE 3:**
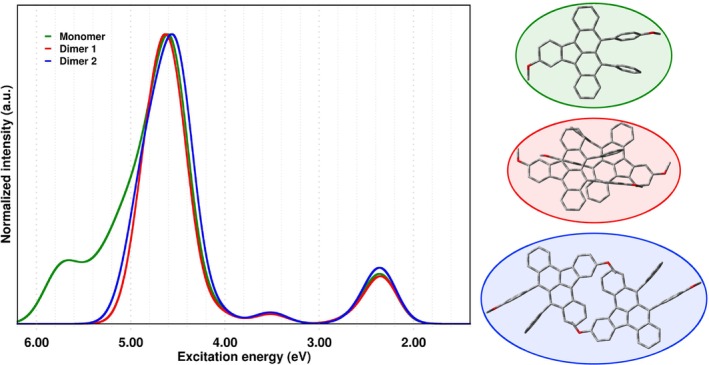
Left panel: UV–Vis simulated absorption spectra computed in gas phase at TD‐CAM‐B3LYP theory levels with a 6‐31+G(d,p) basis set (using Gaussian broadening with a full‐width at half‐maximum of 0.2 eV). The color scheme is reported in the graph inset. Right panel: model systems comprised in our work, Monomer, Dimer 1, Dimer 2 from top to bottom, respectively.

The steady‐state absorption spectrum simulated for *DimethoxyASI* (Figure 3) spans the entire solar spectrum and reveals at least, three absorption bands centered at *ca*: 2.20 eV (563 nm), 3.50 eV (400 nm), and 4.60 eV (269 nm). In this study, we primarily focus on the theoretical characterization of the lowest energy spectral region, corresponding to the two absorption bands below 4 eV.

In Table S2, we report the vertical excitation energies (VEEs) computed for the DimethoxyASI monomer and the two dimeric species (Dimer 1 and Dimer 2) within LR‐TDDFT formalism.

For the refined monomeric structure (see Figure 3 and Figure S1 in the ESI), the first singlet excited state at 2.35 eV is bright (f=0.258) and has a predominantly locally‐excited (LE) nature, where the reorganization of the electron density involves the entire indenotetracene scaffold. This result aligns well with previous experimental and theoretical studies by Cramer and coworkers [54]. At higher energy, the ${\text{S}}_{2}$ state at 2.64 eV is weakly allowed (f=0.034) and exhibits partial charge transfer (CT) character. The $S_{3}\leftarrow S_{0}$ transition (3.51 eV, f=0.055) is characterized by two NTO pairs. The major contribution imparting partial CT character to this transition originates from the electron density on the two phenyl groups (hole) which shifts toward the indenotetracene (electron) once photoexcited. The ${\text{S}}_{4}$ electronic state shows a pronounced CT nature but is optically dark, while ${\text{S}}_{5}-{\text{S}}_{7}$ states above 4.00 eV are described by two NTO pairs with a partial CT character. For the $S_{8}\leftarrow S_{0}$ electronic transition, the dominant hole‐electron pair involves electronic density localized on the two phenyl rings and the indenotetracene core. For the ${\text{S}}_{9}$ and ${\text{S}}_{10}$ states, both hole‐electron pairs primarily exhibit an LE‐type character, also involving the electron density of the two peripheral phenyl rings.

The analysis of VEEs and their contributions to the absorption spectrum, specifically the NTO pairs (see Figure S2), associated with the molecular entity designated as Dimer 1, has yielded several noteworthy observations. The first electronic transition at 2.30 eV exhibits a characteristic localized excitation nature and is weakly bright. Following this, the first low‐energy optically allowed transition at 2.34 eV (${\text{S}}_{2},f=0.410$) retains the same LE properties, with a redistribution of electron density within each individual monomeric unit. Furthermore, electronic transitions ${\text{S}}_{3}$ and ${\text{S}}_{4}$ are computed at 2.65 and 2.67 eV, respectively. Both states are characterized by a single NTO pair with partial intramolecular CT character, similar to the ${\text{S}}_{2}$ state of the Monomer. Conversely, electronic transitions leading to ${\text{S}}_{5}$ and ${\text{S}}_{6}$ are optically dark (f=0.001) and predominantly exhibit an intermolecular CT character, signifying electron density transfer between monomers across the entire molecular scaffold. Meanwhile, the electronic states ${\text{S}}_{7}$ and ${\text{S}}_{8}$ (3.38 eV) retain the same intramolecular CT nature. In this particular case, however, the electron density, initially localized on the two phenyl rings, migrates across the entire indenotetracene framework. Notably, the transitions to ${\text{S}}_{9}$ (f=0.002) and ${\text{S}}_{10}$ (f=0.079) at 3.52 eV are characterized by two hole‐electron pairs, revealing a subtle intramolecular CT character. This behavior manifests as a photoinduced electron density shift from the tetracene moiety toward the indenic component.

Turning to Dimer 2, notable differences emerge in its optical properties. As before, and for consistency, we focus on the first ten singlet electronic transitions (see Figure S3). The first transition (${\text{S}}_{1}$, 2.32 eV) is optically forbidden, while the brightest transition (${\text{S}}_{2}$, f=0.519) is located at 2.35 eV and exhibits a LE character, leading to a redistribution of electron density locally on the indenotetracene scaffold. For the transitions computed at 2.61 eV (${\text{S}}_{3}$, f=0.053) and 2.62 eV (${\text{S}}_{4},f=0.001$), NTO analysis reveals a partial CT character, with weak involvement of the phenyl rings in the electronic reorganization and an intermolecular charge transfer component within each monomer, facilitating electron density flow across the entire scaffold. ${\text{S}}_{5}$ and ${\text{S}}_{6}$ states are isoenergetic, optically forbidden, and exhibit intermolecular CT involving both monomers. Electronic transitions at ${\text{S}}_{7}$ (dark, f=0.003) and ${\text{S}}_{8}$ (weakly bright, f=0.099) occur at 3.51 eV and are described by two hole‐electron pairs, supporting an intramolecular CT nature. At 3.58 eV, the dark transitions ${\text{S}}_{9}$ and ${\text{S}}_{10}$ are described by a single hole‐electron pair, highlighting a charge transfer process from the phenyl rings to the indenotetracene core within both monomeric units.

At higher energy, both dimers exhibit a bright electronic transition between 4 and 5 eV (4.55 eV, f=1.346 for Dimer 1 and 4.50 eV, f=1.788 for Dimer 2), dominated by locally excited states, where electronic density redistribution occurs primarily within each monomeric unit.

In conclusion, the two dimers also exhibit subtle differences in their HOMO‐LUMO energy gaps, which provide important insights into their electronic and structural properties [112, 113, 114, 115]. Dimer 2 shows a slightly larger HOMO‐LUMO gap (4.34 eV, with HOMO at $-0.22494\;{\text{E}}_{h}$ and LUMO at $-0.06516\;{\text{E}}_{h}$) compared to Dimer 1 (4.20 eV, with HOMO at $-0.22475\;{\text{E}}_{h}$ and LUMO at $-0.07022\;{\text{E}}_{h}$), accompanied by a shift in the LUMO energy. This suggests that Dimer 2 has a more stable electronic configuration, with a higher LUMO energy, making it less prone to charge acceptance than Dimer 1. Additionally, the slightly lower HOMO energy in Dimer 2 indicates a stronger binding of the valence electrons, which may be due to less effective π‐π interactions or weaker molecular coupling. These electronic differences reflect the intermolecular interactions within the crystal, likely arising from variations in molecular packing, such as changes in interplanar distance or stacking angles. Consequently, Dimer 1, with its smaller gap, may facilitate easier charge transfer and be more suitable for optoelectronic applications, whereas Dimer 2, with its larger gap, could exhibit more insulating behavior while offering greater electronic stability.

### 3.3 Transition Electric Dipole Moments in the Excited States

As previously discussed, two distinct adducts are present in the crystallographic structure, differing not only in their geometric arrangements but also in key electronic properties, such as the character of the electronic manifold and the transition probabilities. For materials exhibiting polymorphism, it is essential to identify a specific property that enables the selective study of different dimeric forms within a single crystal.

A key property in this context is the magnitude and orientation of the transition electric dipole moment (TEDM, denoted as ${\text{d}}_{nm}$), which determines whether an electronic transition can occur between an initial state, m, and a final state, n. To promote an electron from the ground state to an higher‐energy electronic state two conditions must be satisfied: (i) the photon must deliver the correct amount of energy and (ii) the electric field of the photon must align with the TEDM direction. The TEDM is a complex vector quantity that includes the phase factors associated with the two electronic states. The direction of the TEDM defines the polarization of the transition which, in turn, dictates the outcome of the interaction with an electromagnetic wave with a given polarization. Meanwhile, the magnitude squared, ${\left|d_{nm}\right|}^{2}$, determines the strength of the interaction, reflecting the charge distribution within the molecule.

**FIGURE 4:**
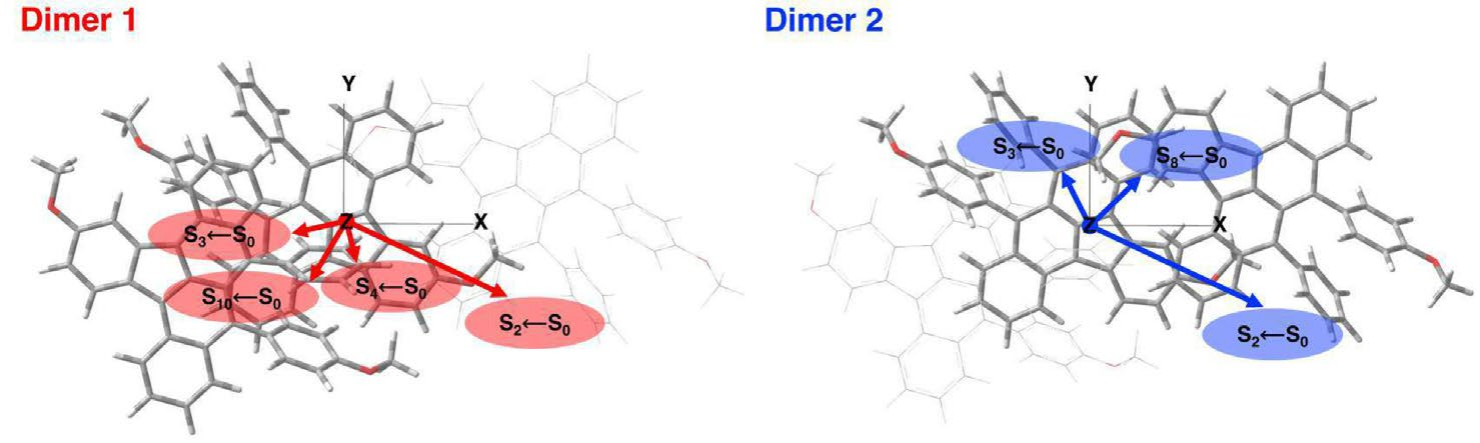
Mutual orientation of the transition electric dipole moments in Dimer 1 (red, left) and Dimer 2 (blue, right) species for allowed (f>0) electronic transitions computed at TD‐CAM‐B3LYP/6‐31+G(d,p) theory level. For better visualization, each TEDM squared magnitude has been multiplied by a factor of 2.5.

A closer examination of these dipole moments and their impact on the crystal's properties can provide valuable insights into its electronic structure and overall behavior. For Dimer 1 and Dimer 2, the TEDMs vector components and the squared magnitude are compared in Table S3 in ESI, with graphical depictions in Figure 4. The TEDMs values, calculated for the two dimeric species, along with their detailed analysis, are presented and discussed here for the first time. In Dimer 1, the TEDMs for the bright electronic transitions are as follows: for ${\text{S}}_{2}$, the values are 2.4144 (x‐axis), −1.0676 (y‐axis), and −0.4247 (z‐axis), resulting in a magnitude of 7.1495. For ${\text{S}}_{3}$, the TEDM has the following components −0.7012,−0.1316 and −0.0058 along the *xyz* axis, respectively, with a magnitude of 0.5090. For ${\text{S}}_{4}$, it is 0.1735 (x‐axis), −0.5200 (y‐axis), and −0.1503 (z‐axis), with a magnitude of 0.3231. Lastly, for ${\text{S}}_{10}$, the dipole moment is −0.4580 (x‐axis), −0.8282 (y‐axis), and −0.1360 (z‐axis), resulting in a magnitude of 0.9142. For Dimer 2 only the electronic transitions $S_{2,3,8}\leftarrow S_{0}$ are allowed and the corresponding TEDMs are: for ${\text{S}}_{2}$, we compute 2.6920 (x‐axis), −1.2700 (y‐axis), and −0.3820 (z‐axis) and a magnitude of 9.0057. For ${\text{S}}_{3}$, it is −0.3974 (x‐axis), 0.7784 (y‐axis), and 0.2485 (z‐axis), with a magnitude of 0.8256. Finally, for ${\text{S}}_{8}$, the TEDM is 0.7588 (x‐axis), 0.7285 (y‐axis), and 0.22267 (z‐axis), resulting in a magnitude of 1.1580. In summary, the observation of distinct transition electric dipole moments for the two dimers suggests that they may exhibit unique behaviors in their interactions with light radiation. This finding has profound implications for their electronic properties, including electrical conductivity, and offers a deeper understanding of how polymorphism can influence the optical and electronic performance of organic semiconductor materials.

### 3.4 Characterization of Triplet State Energies

In this study, we primarily focused on the characterization of the lowest energy triplet states to gain insights into the potential for singlet fission in the examined models. At this stage, our attention is directed solely toward verifying the energy condition for singlet fission, that is, whether $\text{E}({\text{S}}_{1})\geq 2\text{E}({\text{T}}_{1})$ is satisfied.

The calculation of triplet state energies has been conducted using different approaches. In the case of the Monomer, the first variational triplet state's energy, calculated as a $\Delta_{SCF}$ energy, is found to be at 1.24 eV, a value corroborated by the wavefunction stability analysis for the T1 state. However, the triplet vertical excitation energy calculated via LR‐TDDFT appears to be significantly lower (0.44 eV). On the contrary, the TDDFT calculation performed within the Tamm‐Dancoff approximation rectifies this issue, with only a 0.01 eV discrepancy compared to the previous cases. The calculation of T1 via $\Delta_{\text{SCF}}$ for Dimer 1 indicates that the single determinant for the T1 state is not stable (2.14 eV), yielding an erroneous value, as demonstrated by the wavefunction stability analysis, which returns a more physically plausible value of 1.24 eV. In LR‐TDDFT calculations, T1 is found to be situated at 0.46 eV, nearly degenerate with T2 (0.47 eV), whereas in the Tamm‐Dancoff approximation, the energy of both T1 and T2 states is determined to be 1.25 eV. Finally, for Dimer 2, there are no wavefunction instabilities for the T1 state, and consequently, in both approaches ($\text{T}1_{\Delta_{\text{SCF}}}$ e $\text{T}1_{\text{Stable}}$), it is calculated at 1.24 eV. Similar to the previous cases, LR‐TDDFT calculations significantly underestimate the energy of the triplet states (0.44 eV for both T1 and T2 states). In contrast, the TDDFT/TDA approach within the Coulomb‐attenuated functional has proven to be suitable, yielding both values at 1.25 eV.

### 3.5 Identification and Assignment of Raman Active Vibrational Modes

Since both dimers share the same chemical identity, the intramolecular vibrations (in the 800–$1700\;{\text{cm}}^{-1}$ range) can be confidently assigned based on the refined crystallographic structure of the isolated monomer (see Figure S4, green curve, top panel). The corresponding normal modes of vibration are reported in the supporting information under Figure S5. The vibrational mode at $831\;{\text{cm}}^{-1}$ involves the twisting of the HC‐CH pattern localized on the indenotetracene and the peripheral phenyl ring, at $879\;{\text{cm}}^{-1}$ appear the ring breathing motion of the indenotetracene core. At higher energy, the trigonal stretching mode of the phenyl ring is present at $992\;{\text{cm}}^{-1}$; slightly above, at $1010\;{\text{cm}}^{-1}$, the asymmetric C‐C stretching coupled to the scissoring of the‐CH bonds are founded. The torsion of both $-{\text{CH}}_{3}$ groups are degenerate and located at 1129, $1130\;{\text{cm}}^{-1}$. HC‐CH scissoring modes coupled to methyl torsion $\left(1165\;{\text{cm}}^{-1}\right)$ or else to C‐C and C‐O stretching $\left(1182\;{\text{cm}}^{-1}\right)$ come first two asymmetric stretching modes involving the skeletal rings vibrations (1291 and $1369\;{\text{cm}}^{-1})$. In the spectral region above $1400\;{\text{cm}}^{-1}$ more complex vibrational modes compositions involving C‐C bonds of the indenotetracene scaffold, the scissoring of methyl groups and in plane deformations of HC‐CH are observed at 1409 and $1436\;{\text{cm}}^{-1}$. Well defined asymmetric C=C stretching modes localized on the indenotetracene plane are computed at $1534\;{\text{cm}}^{-1}$. The asymmetric C=C stretching belonging to the *p*‐methoxybenzyl substituent is computed at $1556\;{\text{cm}}^{-1}$, while the frequency computed at $1605\;{\text{cm}}^{-1}$ originates from the asymmetric C=C stretching motion of the peripheral six‐membered rings of the indenotetracene unit.

**FIGURE 5:**
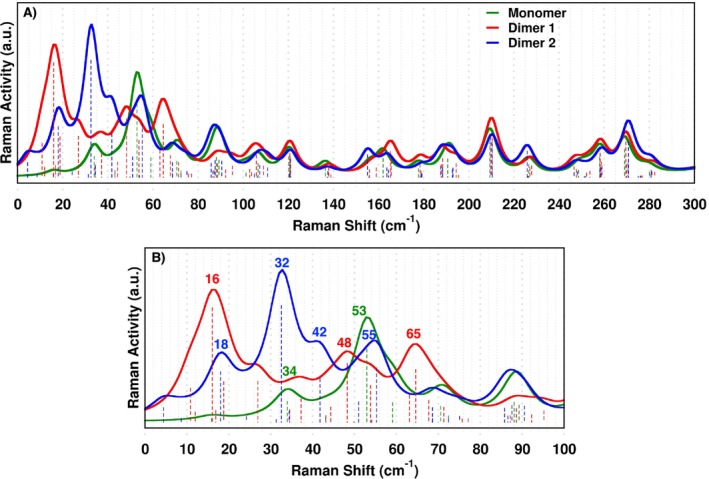
Panel A: Raman spectrum within $300\;{\text{cm}}^{-1}$ range, computed for the refined crystallographic structures of the Monomer (green curve), Dimer 1 (red curve), and Dimer 2 (blue curve) in the ground state at the B3LYP/6‐31+G(d,p) level of theory. Panel B: detailed view of the low‐frequency region. Raman activities are reported in arbitrary units (a.u.) and normalized.

The Raman vibrational spectra of both refined dimeric structures in the ground state exhibit a similar profile in terms of shape and mode composition within the ∼800–$1700\;{\text{cm}}^{-1}$ range (Table 1). For completeness, these spectra are presented in Figure S4, central and bottom panels. On the contrary, subtle differences are found in the phononic spectral region populated by the *intermolecular* vibrational modes such as collective translational and rotational motions which affect the dynamical deformation of the crystal lattice. Lattice phonons, involving very low frequency Raman shift (5–$150\;{\text{cm}}^{-1}$, known as the THz‐Raman region), act as probes of the diversity of the chemical environment being highly sensitive to intermolecular forces and different molecular packing [116, 117, 118, 119, 120, 121]. The Raman spectrum region corresponding to low‐frequency collective modes, is presented in Figure 5.

**TABLE 1:** Harmonic Raman frequencies for *DimethoxyASI* (second column) and their scaled values (third column), computed for the crystallographic monomer in the ground state at the B3LYP/6‐31+G(d,p) level of theory. The fourth column provides the proposed vibrational mode assignments. The composition of the vibrational modes is illustrated in Figure S5.

Mode	Harm. freq.	Scaled freq.^a^	Proposed assignment
1.	862	831	indenotetracene τ HC‐CH + phenyl τ HC‐CH
2.	911	879	indenotetracene rings breathing
3.	1029	992	ν trigonal phenyl ring
4.	1048	1010	asym ν C‐C + δ HC‐CH
5.	1171, 1172	1129, 1130	$-{\text{CH}}_{3}$ torsion
6.	1208	1165	δ HC‐CH + $-{\text{CH}}_{3}$ torsion
7.	1226	1182	δ HC‐CH + ν C‐C + ν (${\text{C}}_{Ph}$‐${\text{OCH}}_{3}$)
8.	1339	1291	asym ν rings
9.	1420	1369	asym ν rings
10.	1462	1409	δ $-{\text{CH}}_{3}$ + ρ HC‐CH + ν C‐C on tetracene core
11.	1489	1436	δ $-{\text{CH}}_{3}$ + δ HC‐CH + ν C‐C on tetracene core
12.	1591	1534	ρ,δ HC‐CH + ν C‐C on indenotetracene core
13.	1614	1556	asym ν C‐C on Ph‐${\text{OCH}}_{3}$ ring
14.	1666	1605	asym ν C‐C on peripheral indenotetracene rings

^a^ Scaling factor: 0.9642. All frequency values are reported in wavenumbers $\left({\text{cm}}^{-1}\right)$. Vibrational mode descriptors: τ (twisting), δ (scissoring), ρ (rocking), ν (stretching), asym (asymmetric), Ph (phenyl).

The marked differences in the lattice phonon modes below $80\;{\text{cm}}^{-1}$ between the two dimers highlight the critical role of intermolecular interactions in shaping the vibrational response of *DimethoxyASI* crystals. Below we discuss our results focusing on bands with non‐negligible Raman intensity and possibly exhibiting little overlap between the three models. For the Monomer case, two bands at 34 and $53\;{\text{cm}}^{-1}$ can be identified, corresponding to the π‐stacking of the peripheral phenyl rings and a combined motion involving out‐of‐plane bending of the indenotetracene and scissoring of the phenyl rings, respectively (see Figure S6). Dimer 1 shows mainly three bands: the most intense one is located at $16\;{\text{cm}}^{-1}$ followed by 48 and $65\;{\text{cm}}^{-1}$, the latter being nearly isoenergetic. From the visual inspection of the normal coordinates, these modes are identified as the combination of several collective contributions, see Table 2. A comparison of Figure 5 reveals a vibrational mode that serves as a key spectroscopic marker for distinguishing the two structural domains within the same crystallite. Notably, the Raman feature at $32\;{\text{cm}}^{-1}$, uniquely active for Dimer 2, stands out as a definitive spectroscopic fingerprint, enabling the precise identification of distinct structural domains within the same crystallite. The remaining modes have been also characterized and their description is reported in Table 2. In conclusion, the spectra in Figure 5 and Figure S4 confirm that above $100\;{\text{cm}}^{-1}$ the vibrational patterns of the three models exhibit strong similarities, further reinforcing the validity of monomer‐based calculations as a reliable framework for interpreting the experimental Raman spectra for the 800–$1700\;{\text{cm}}^{-1}$ region.

**TABLE 2:** Harmonic frequencies (in ${\text{cm}}^{-1}$) of the lattice phonon modes computed for the three models along with an assignment proposal of the normal modes displacement vectors. Descriptors for normal modes of vibration: τ twisting, δ scissoring, π weak dispersion interactions.

	Harm. freq.	Scaled freq.^a^	Proposed assignment
**Monomer**	34	32	phenyl rings π‐stack
	53	49	τ indenotetracene + δ phenyl rings
**Dimer 1**	16	15	π‐stack + δ phenyl rings
	48	45	π‐stack + τ indenotetracene
	65	60	τ indenotetracene
**Dimer 2**	18	17	π‐stack + δ phenyl rings
	32	30	π‐stack
	42	39	δ phenyl rings
	55	52	π‐stack + τ indenotetracene

^a^ Scaling factor: 0.9642. See Figure S6 for details.

## 4 Conclusions

In this work, we provided a detailed DFT/TDDFT computational characterization of an asymmetrically dimethoxy‐substituted diarylindenotetracene derivative, offering new insights into its structural, electronic, and vibrational properties. Our analysis of the crystal structure, based on previously reported X‐ray diffraction data, identified for the first time the presence of two distinct dimeric arrangements, whose electronic interactions lead to differing optical responses. TDDFT calculations revealed how these dimeric configurations influence electronic transitions, particularly in terms of local excitation and charge‐transfer contributions. The computation of transition dipole moments further suggested that selective excitation of one dimer over the other could be achieved, which is particularly relevant in the context of single‐crystal polymorphism. Furthermore, our investigation of the lowest energy triplet states provided insights into the potential for singlet fission in this system. By comparing different computational approaches, we identified ΔSCF and TDA as the most reliable methods for accurately describing triplet state energies, ensuring a robust assessment of the singlet fission criterion. DFT calculations enabled a detailed assignment of vibrational features, including key low‐frequency intermolecular modes that are highly sensitive to the different crystalline environments experienced by the two dimers. The analysis of phonon lattice modes demonstrates the ability of vibrational spectroscopy to distinguish structural domains within the same crystal, highlighting its capability to resolve polymorphic mixtures at the molecular level. Overall, this study enhances our understanding of the relationship between molecular packing, electronic structure, and optical properties in indenotetracene derivatives. The insights gained here contribute to the broader exploration of singlet fission materials and underscore the crucial role of theoretical approaches within the DFT/TDDFT framework in the design and characterization of novel organic semiconductors.

## Author Contributions

**Federico Coppola:** conceptualization, methodology, data curation, formal analysis, investigation, writing – original draft, writing – review and editing; **Raoul Carfora:** formal analysis, investigation, writing – original draft, writing – review and editing; **Nadia Rega:** conceptualization, funding acquisition, writing – review and editing. All authors have read and agreed to the published version of the manuscript.

## Conflicts of Interest

The authors declare no conflicts of interest.

## Supporting information




**Data S1.** Electronic Supporting Information (ESI) available: Convergence thresholds used for the ground state optimizations. Excitation energies and oscillator strengths for singlet and triplet states of monomeric and dimeric structures. Natural transition orbitals for monomeric and dimeric structures. Ground‐to‐excited state transition dipole moments for the first ten singlet states of Dimer 1 and Dimer 2. Infrared and Raman Spectra computed for the monomer, Dimer 1, and Dimer 2 in the ground state. Normal mode displacement vectors corresponding to Raman peaks between 800–$1600\;{\text{cm}}^{-1}$ and below $300\;{\text{cm}}^{-1}$. Equilibrium geometries of monomeric and dimeric structures.

## Data Availability

The data supporting this article have been included as part of the Supplementary Information.
