# Supplementary material for: Electronic Structure and Vibrational Properties of Indenotetracene‐Based Crystal
Source: J Comput Chem. 2025 May 24;46(14):e70141. doi: 10.1002/jcc.70141 (PMC12102687; doi:10.1002/jcc.70141)
Supplement: Supplementary file 1 — Data S1. Electronic Supporting Information (ESI) available: Convergence thresholds used for the ground state optimizations. Excitation energies and oscillator strengths for singlet and triplet states of monomeric and dimeric structures. Natural transition orbitals for monomeric and dimeric structures. Ground‐to‐excited state transition dipole moments for the first ten singlet states of Dimer 1 and Dimer 2. Infrared and Raman Spectra computed for the monomer, Dimer 1, and Dimer 2 in the ground state. Normal mode displacement vectors corresponding to Raman peaks between 800–1600cm−1 and below 300cm−1. Equilibrium geometries of monomeric and dimeric structures. [file JCC-46-0-s001.pdf]

Electronic Supporting Information

Electronic Structure and Vibrational Properties of Indenotetracene-Based Crystal†

Federico Coppola,[*,†] Raoul Carfora,[†,‡] and Nadia Rega[*,‡,†,¶]

†*Scuola Superiore Meridionale, Largo San Marcellino 10, I-80138, Napoli, Italy.*

‡*Department of Chemical Sciences, University of Napoli Federico II, Complesso Universitario di M.S. Angelo, via Cintia 21, I-80126, Napoli, Italy.*

¶*Istituto Nazionale Di Fisica Nucleare, sezione di Napoli, Complesso Universitario di M.S. Angelo ed. 6, via Cintia 21, I-80126, Napoli, Italy.*

E-mail: f.coppola@ssmeridionale.it; nadia.rega@unina.it

S1 Contents

Table S1: Converge criteria applied for the ground state optimizations

Table S2: Vertical excitation energies and oscillator strengths computed for singlet and triplet states on the monomeric and dimeric structures

Figure S1: NTOs for the *hole-electron* transition of the first ten singlet excited states calculated for Monomer

Figure S2: NTOs for the *hole-electron* transition of the first ten singlet excited states calculated for Dimer 1

Figure S3: NTOs for the *hole-electron* transition of the first ten singlet excited states calculated for Dimer 2

Table S3: Ground to excited state transition electric dipole moments computed for the first ten singlet electronic states for Dimer 1 and Dimer 2

Figure S4: Infrared and Raman spectrum computed on the refined crystallographic structure of Monomer, Dimer 1, and Dimer 2 in the ground state

Figure S5: Normal modes displacement vectors associated to the Raman frequencies between 800-1600 cm^{-}1

Figure S6: Normal modes displacement vectors associated to the Raman frequencies below 300 cm^{-}1

Table S4: Cartesian coordinates of the *DimethoxyASI* Monomer

Table S5: Cartesian coordinates of the *DimethoxyASI* Dimer 1

Table S6: Cartesian coordinates of the *DimethoxyASI* Dimer 2

Table S1. Converge criteria applied for the ground state optimizations of Monomer, Dimer 1, and Dimer 2 geometries at B3LYP/6-31+G(d,p) theory level.

Threshold criteria	**Monomer**	**Dimer 1**	**Dimer 2**
Max. Force: 0.000450 Hartree/Bohr	0.000011	0.000055	0.000005
RMS Force: 0.000300 Hartree/Bohr	0.000002	0.000007	0.000001
Max. Displacement: 0.001800 Bohr	0.000671	0.001067	0.000812
RMS Displacement: 0.001200 Bohr	0.000107	0.000159	0.000109

Table S2. Vertical Excitation Energies (in eV) and oscillator strengths (f, dimensionless) computed for singlet and triplet states at TD-CAM-B3LYP/6-31+G(d,p) level on the monomeric and dimeric structures optimized in the ground state at B3LYP/6-31+G(d,p) theory level.

E.S.	Monomer		Dimer 1		Dimer 2	
$T1_{\Delta SCF}$	1.24	–	2.14	–	1.24	–
$T1_{Stable}$	1.24	–	1.24	–	1.24	–
$T1_{TDA}$	1.25	–	1.25	–	1.25	–
S1	2.35	(0.258)	2.30	(0.010)	2.32	(0.000)
S2	2.64	(0.034)	2.34	(0.410)	2.35	(0.519)
S3	3.51	(0.055)	2.65	(0.033)	2.61	(0.053)
S4	3.58	(0.003)	2.67	(0.021)	2.62	(0.001)
S5	4.05	(0.040)	2.98	(0.001)	3.19	(0.000)
S6	4.21	(0.021)	3.21	(0.001)	3.20	(0.000)
S7	4.28	(0.065)	3.38	(0.003)	3.51	(0.003)
S8	4.48	(0.108)	3.38	(0.004)	3.51	(0.099)
S9	4.54	(0.940)	3.52	(0.002)	3.58	(0.002)
S10	4.59	(0.026)	3.52	(0.079)	3.58	(0.003)
S11	4.61	(0.151)	3.64	(0.000)	3.68	(0.001)
S12	4.75	(0.090)	3.88	(0.000)	3.68	(0.001)
S13	4.77	(0.346)	3.97	(0.000)	4.03	(0.002)
S14	4.80	(0.199)	4.03	(0.024)	4.04	(0.067)
S15	4.97	(0.039)	4.03	(0.039)	4.20	(0.046)
S16	5.00	(0.110)	4.16	(0.001)	4.20	(0.001)
S17	5.03	(0.105)	4.20	(0.012)	4.24	(0.000)
S18	5.06	(0.086)	4.20	(0.009)	4.25	(0.070)
S19	5.10	(0.061)	4.23	(0.022)	4.45	(0.265)
S20	5.15	(0.095)	4.23	(0.019)	4.45	(0.012)
S21	5.23	(0.003)	4.31	(0.021)	4.49	(0.007)
S22	5.30	(0.034)	4.31	(0.031)	4.50	(1.788)
S23	5.34	(0.125)	4.36	(0.002)	4.58	(0.003)
S24	5.36	(0.017)	4.46	(0.112)	4.58	(0.060)
S25	5.40	(0.011)	4.47	(0.027)	4.61	(0.011)
S26	5.47	(0.010)	4.48	(0.096)	4.63	(0.188)
S27	5.53	(0.051)	4.55	(1.346)	4.69	(0.000)
S28	5.56	(0.009)	4.59	(0.091)	4.71	(0.119)
S29	5.57	(0.004)	4.61	(0.223)	4.71	(0.028)
S30	5.61	(0.006)	4.72	(0.253)	4.76	(0.444)
S31	5.66	(0.053)	4.73	(0.045)	4.76	(0.000)
S32	5.66	(0.020)	4.75	(0.095)	4.77	(0.028)
S33	5.68	(0.030)	4.76	(0.164)	4.77	(0.007)
S34	5.72	(0.018)	4.77	(0.229)	4.78	(0.461)
S35	5.76	(0.005)	4.80	(0.278)	4.85	(0.009)
S36	5.78	(0.123)	4.82	(0.008)	4.86	(0.000)
S37	5.81	(0.018)	4.86	(0.044)	4.92	(0.005)
S38	5.83	(0.011)	4.87	(0.318)	4.93	(0.554)
S39	5.86	(0.005)	4.92	(0.015)	4.96	(0.000)
S40	5.89	(0.010)	4.94	(0.006)	4.96	(0.059)

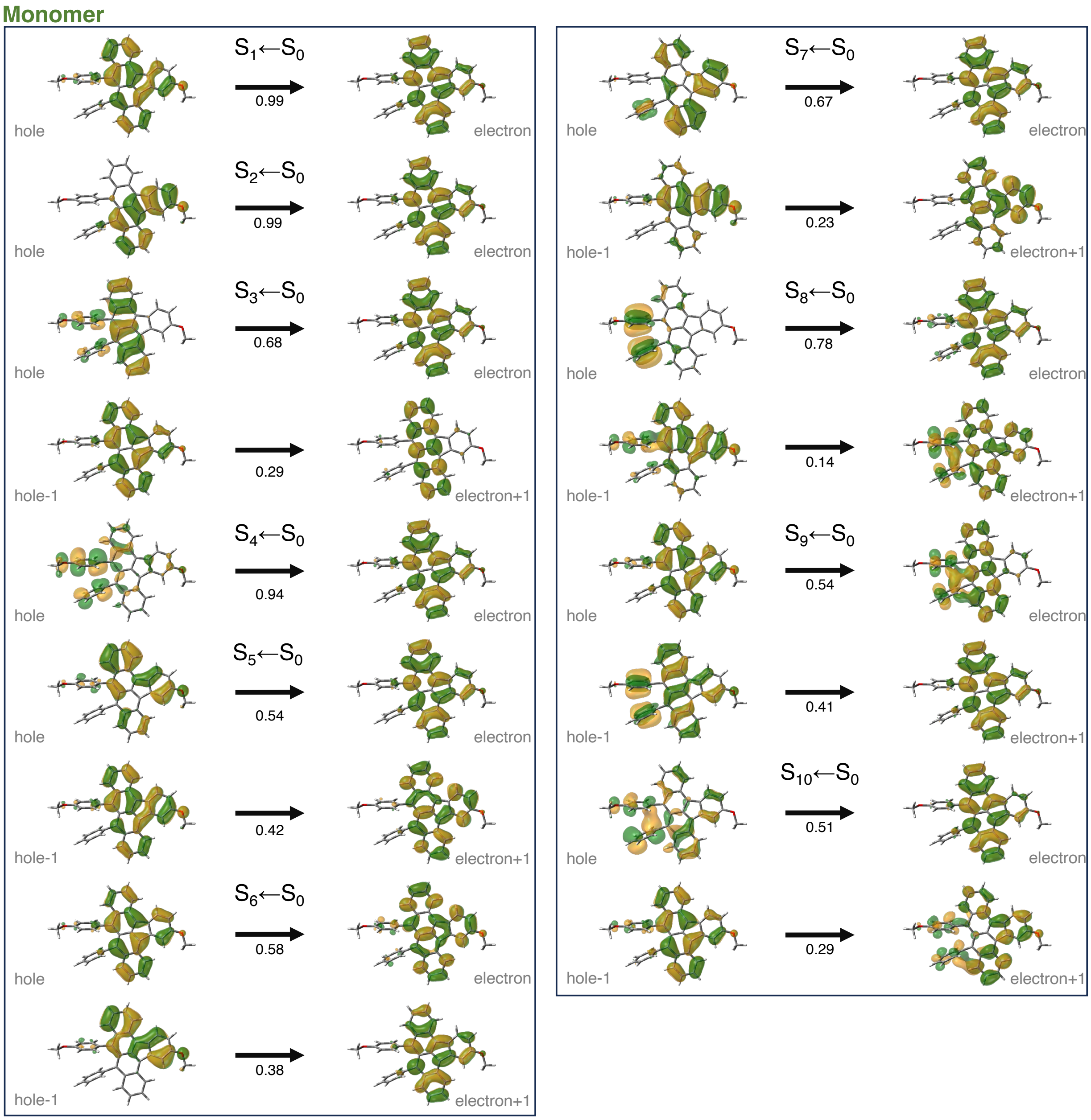

Figure S1. NTOs for the *hole-electron* transition of the first ten singlet excited states that contribute more than 20% calculated for Monomer case with the CAM-B3LYP functional. The iso-surface value=0.02 e/Bohr.

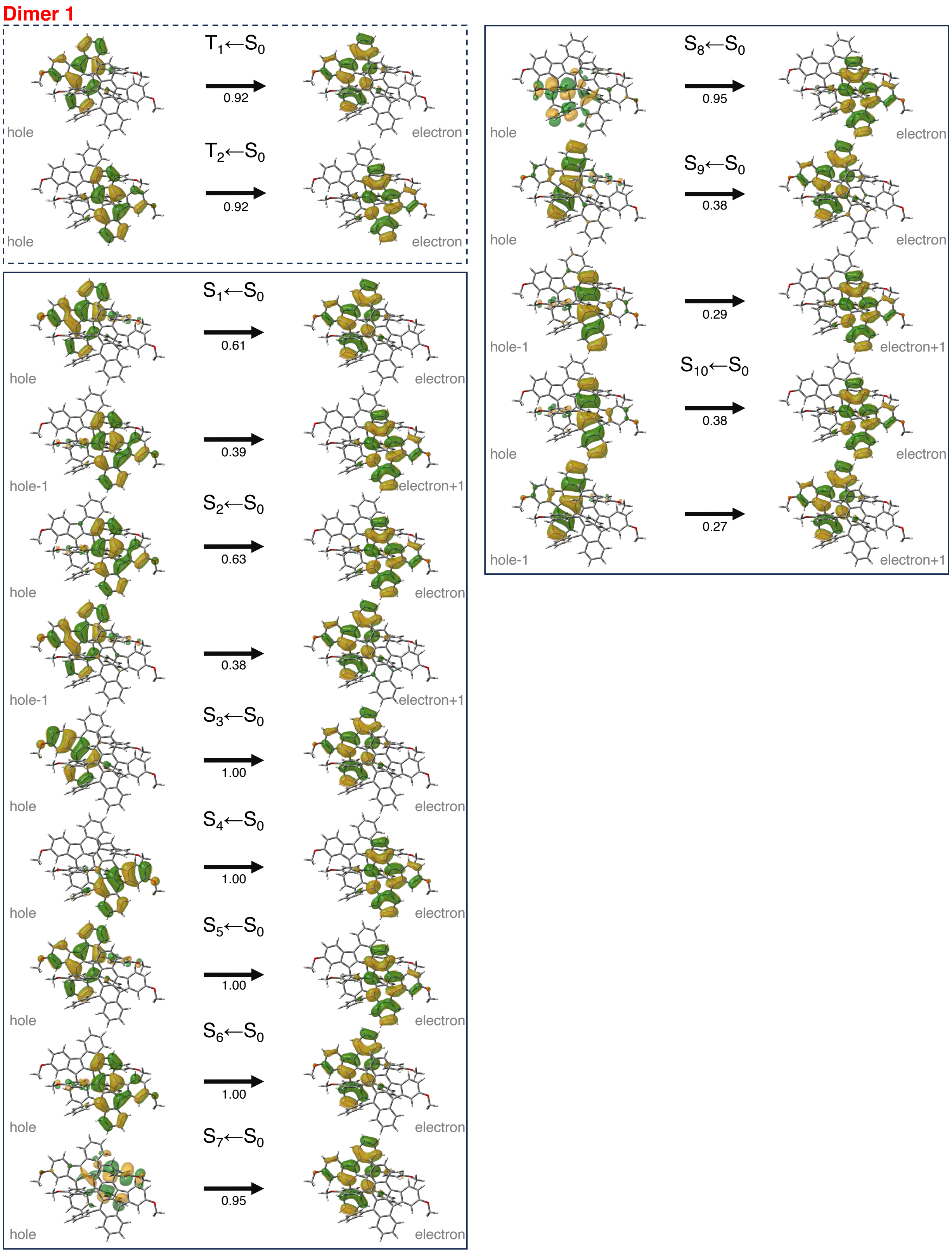

Figure S2. NTOs for the *hole-electron* transition of the first ten singlet excited states that contribute more than 20% calculated for Dimer 1 case with the CAM-B3LYP functional. The iso-surface value=0.02 e/Bohr.

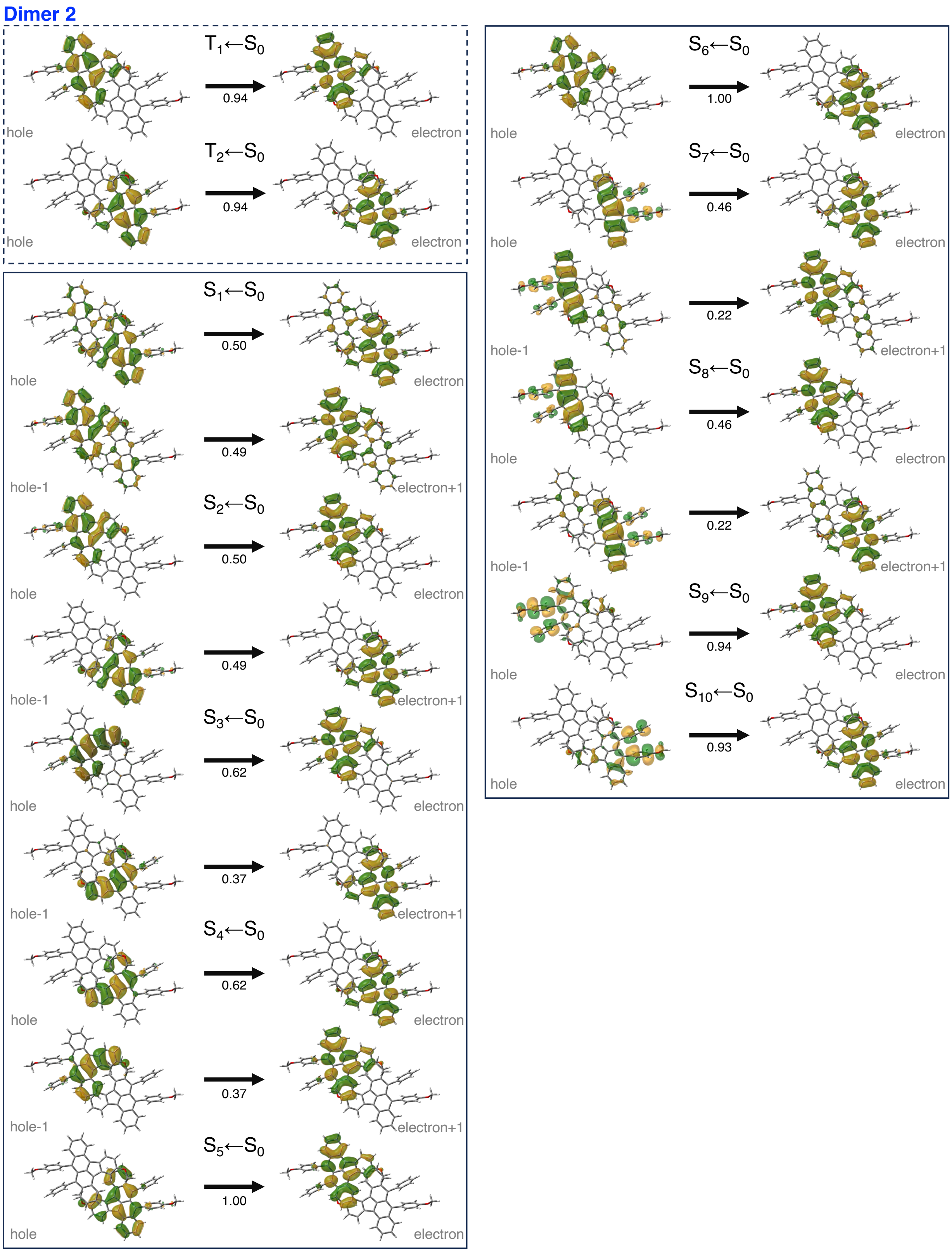

Figure S3. NTOs for the *hole-electron* transition of the first two triplet and first ten singlet excited states that contribute more than 20% calculated for Dimer 2 case with the CAM-B3LYP functional. The iso-surface value=0.02 e/Bohr.

Table S3. Ground to excited state transition electric dipole moments ($|d_{S_0-S_n}|^2$, in atomic units) and relative x, y, z components computed for the first ten singlet electronic states at TD-CAM-B3LYP/6-31+G(d,p) theory level for Dimer 1 and Dimer 2.

	Dimer 1				Dimer 2			
E.S.	x	y	z	$\lvert d_{S_0-S_n}\rvert^2$	x	y	z	$\lvert d_{S_0-S_n}\rvert^2$
S1	0.3981	0.1193	0.0502	0.1753	0.0095	-0.0017	0.0005	0.0001
S2	2.4144	-1.0676	-0.4247	**7.1495**	2.6920	-1.2700	-0.3820	**9.0057**
S3	-0.7012	-0.1316	-0.0058	**0.5090**	-0.3974	0.7784	0.2485	**0.8256**
S4	0.1735	-0.5200	-0.1503	**0.3231**	-0.0512	0.0985	0.0310	0.0133
S5	-0.0671	0.0652	-0.0284	0.0096	-0.0466	0.0065	-0.0083	0.0023
S6	0.0021	0.0866	-0.0463	0.0097	0.0507	-0.0071	0.0090	0.0027
S7	0.1326	-0.1231	-0.0891	0.0407	0.1395	0.1328	0.0413	0.0388
S8	-0.1765	-0.1016	-0.0971	0.0509	0.7588	0.7285	0.2267	**1.1580**
S9	0.1006	-0.0939	-0.0278	0.0197	-0.0438	0.1570	-0.0228	0.0271
S10	-0.4580	-0.8282	-0.1360	**0.9142**	0.0540	-0.1787	0.0228	0.0354

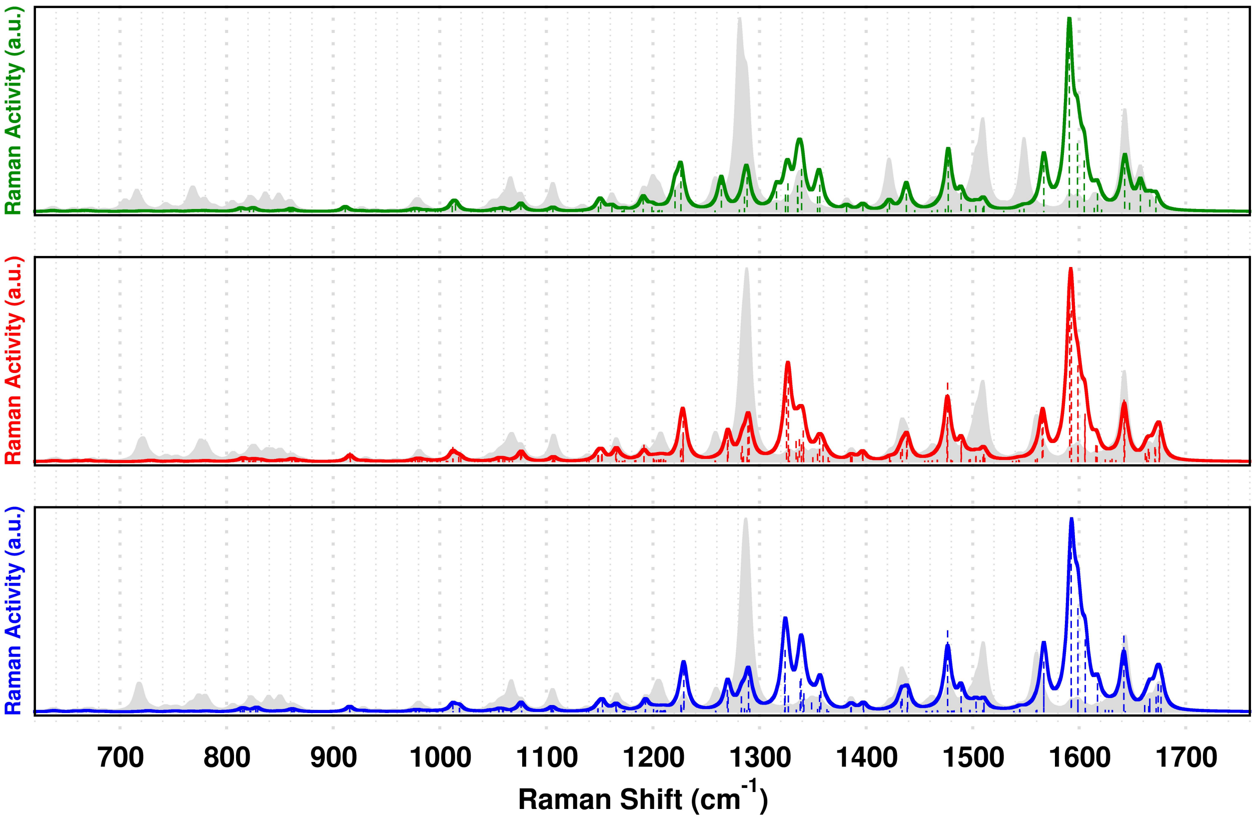

Figure S4. Infrared (gray) and Raman spectrum computed on the refined crystallographic structure of Monomer (top, green curve), Dimer 1 (center, red curve), and Dimer 2 (bottom, blue curve) in the ground state at B3LYP/6-31+G(d,p) theory level. Infrared intensity and Raman activity are reported in arbitrary units (a.u.) and normalized.

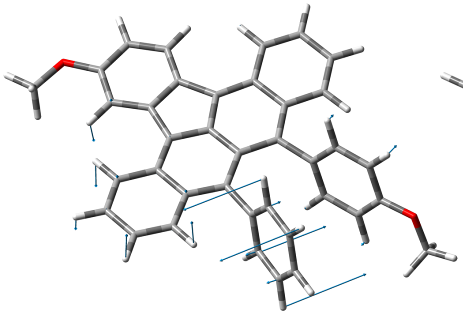

1) 831 cm^{-1}, indenotetracene τ HC-CH + phenyl τ HC-CH

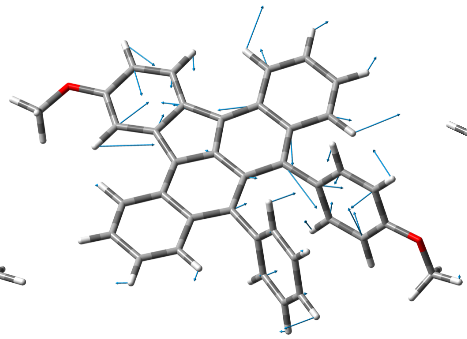

2) 879 cm^{-1}, indenotetracene rings breathing

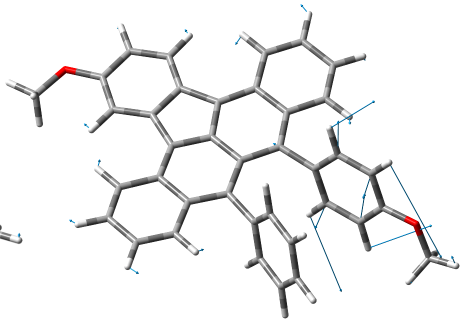

3) 992 cm^{-1}, ν trigonal phenyl ring

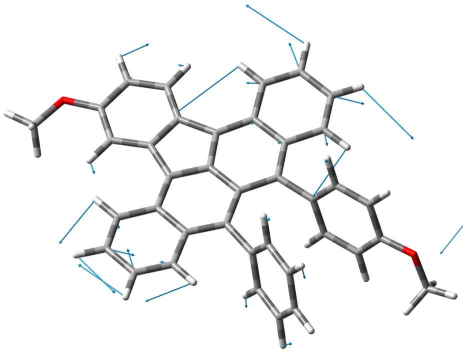

4) 1010 cm^{-1}, asym ν C-C + δ HC-CH

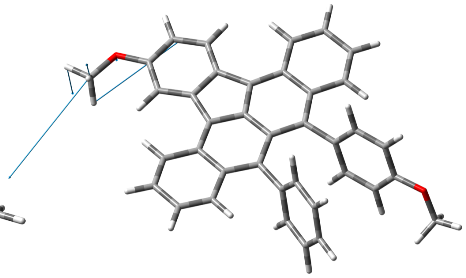

5) 1129 cm^{-1}, -CH_3 torsion

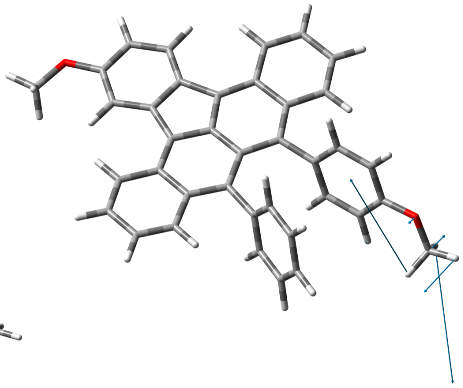

5) 1130 cm^{-1}, -CH_3 torsion

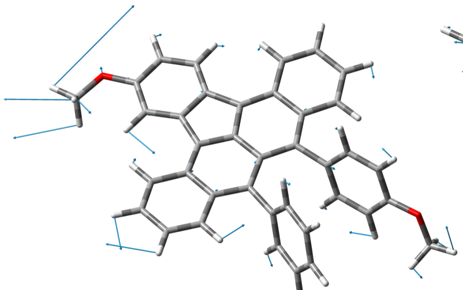

6) 1165 cm^{-1}, δ HC-CH + -CH_3 torsion

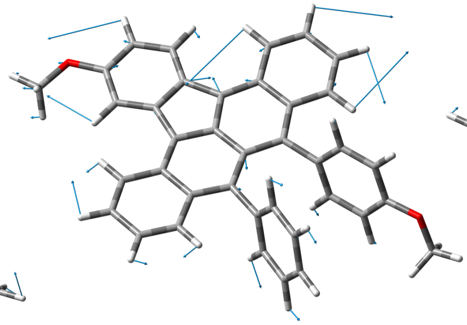

7) 1182 cm^{-1}, δ HC-CH + ν C-C + ν (C_{Ph}-OCH_3)

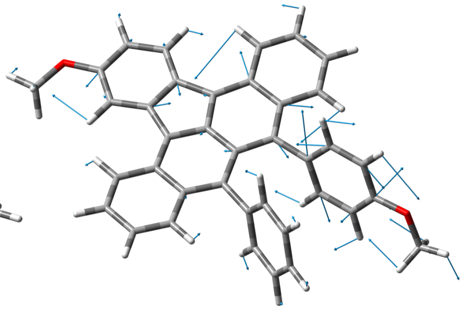

8) 1291 cm^{-1}, asym ν rings

Figure S5. Normal modes displacement vectors computed at B3LYP/6-31+G(d,p) level associated to the Raman frequencies reported in Tab. 1. Descriptors for normal modes of vibration: τ twisting, δ scissoring, ρ rocking, ν stretching.

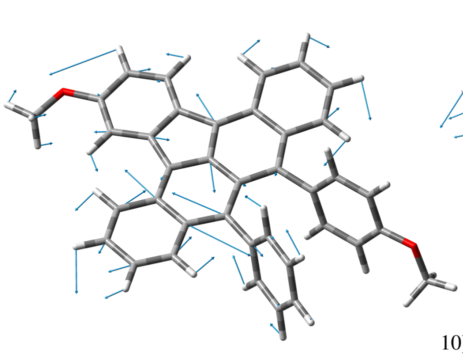

9) 1369 cm^{-1}, asym ν rings

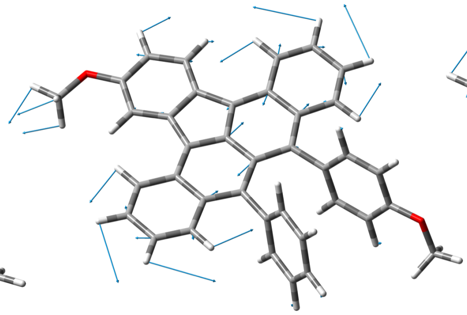

10) 1409 cm^{-1}, δ -CH_3 + ρ HC-CH + ν C-C on tetracene core

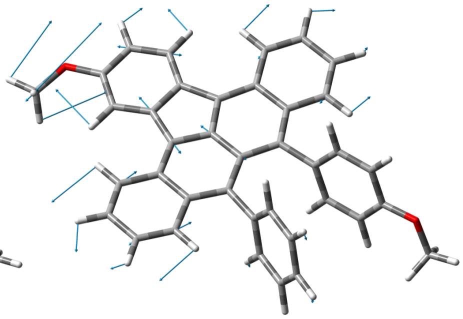

11) 1436 cm^{-1}, δ -CH_3 + δ HC-CH + ν C-C on tetracene core

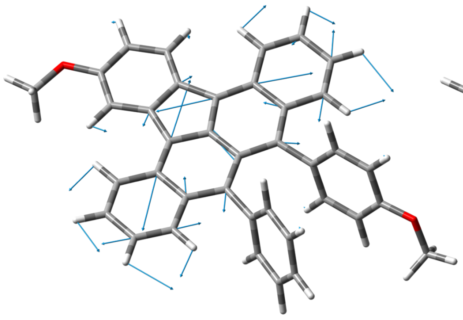

12) 1556 cm^{-1}, asym ν C-C on Ph-OCH_3 ring

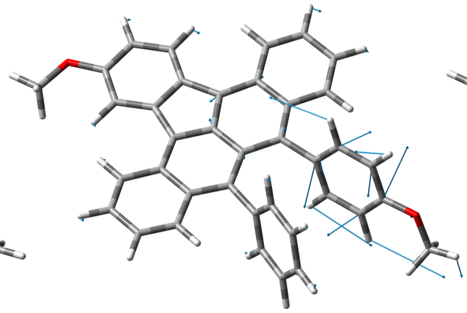

13) 1606 cm^{-1}, ν C-C on peripheral phenyl rings

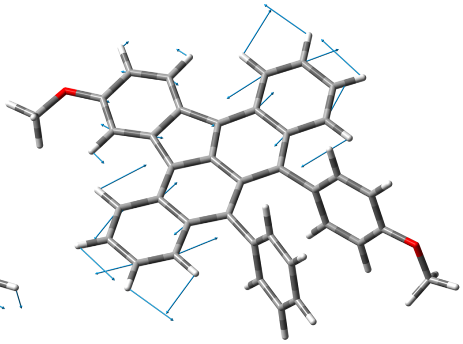

14) 1605 cm^{-1}, asym ν C-C on peripheral indenotetracene rings

Figure S5. Normal modes displacement vectors computed at B3LYP/6-31+G(d,p) level associated to the Raman frequencies reported in Tab. 1 (*cont.*). Descriptors for normal modes of vibration: τ twisting, δ scissoring, ρ rocking, ν stretching.

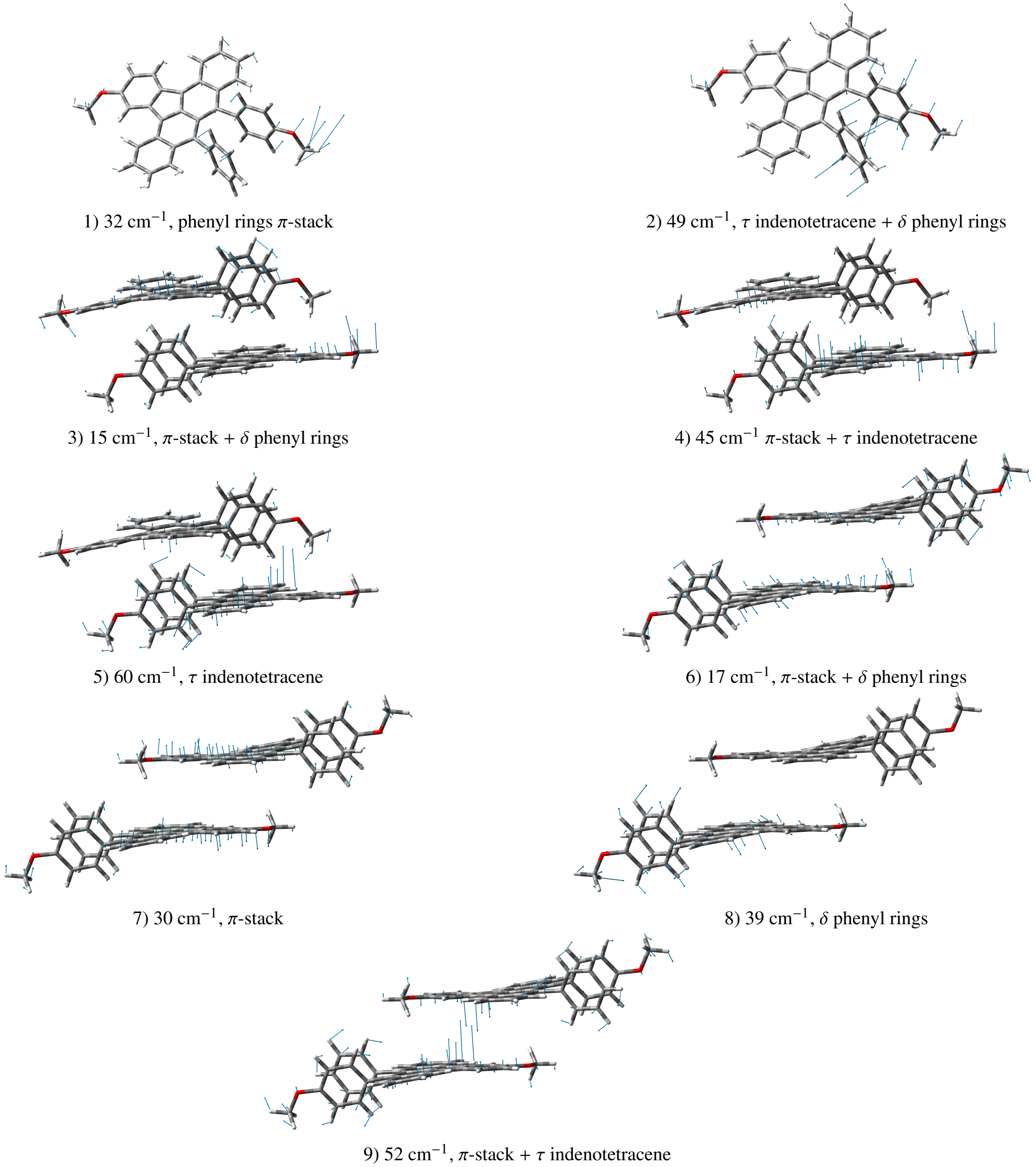

1) 32 cm^{-1}, phenyl rings π-stack

2) 49 cm^{-1}, τ indenotetracene + δ phenyl rings

3) 15 cm^{-1}, π-stack + δ phenyl rings

4) 45 cm^{-1} π-stack + τ indenotetracene

5) 60 cm^{-1}, τ indenotetracene

6) 17 cm^{-1}, π-stack + δ phenyl rings

7) 30 cm^{-1}, π-stack

8) 39 cm^{-1}, δ phenyl rings

9) 52 cm^{-1}, π-stack + τ indenotetracene

Figure S6. Normal modes displacement vectors computed at B3LYP/6-31+G(d,p) level associated to the Raman frequencies reported in Tab. 2.

S2 Equilibrium geometries in cartesian coordinates (Å) at B3LYP/6-31+G(d,p)

Table S4. Minimum energy structure of *DimethoxyASI* Monomer
(nuclear repulsion energy: 4200.8838590017 Hartrees)

C	1.19504	3.6804	0.12952	C	-0.15	-3.74751	0.16458
H	2.26754	3.55617	0.0571	H	0.89799	-4.00688	0.22463
C	0.67257	4.94396	0.19743	C	0.46597	-1.33596	0.07695
H	1.33472	5.80473	0.18174	C	3.09744	0.79883	-1.13702
C	-0.73603	5.13652	0.28656	H	2.52411	0.52617	-2.01782
H	-1.1409	6.14173	0.36128	C	-1.08042	-4.75158	0.18759
C	-1.57852	4.05754	0.26672	H	-0.75789	-5.78625	0.25708
H	-2.64532	4.21858	0.33232	C	-2.87899	-3.13874	0.07362
O	6.61702	1.20824	-0.06012	H	-3.93742	-2.92593	0.07544
C	0.36069	2.50632	0.14532	C	-2.3096	-0.70188	-0.00625
C	-3.3573	1.39493	0.01045	C	2.6259	-1.85744	1.28732
C	0.0861	0.04647	0.07984	C	-2.46829	-4.44323	0.14147
C	-3.61089	-0.00668	-0.05741	H	-3.20256	-5.24303	0.17653
C	2.42666	1.15238	0.03624	C	4.45096	-2.99168	0.16667
C	5.2501	1.16794	-0.09608	H	5.43749	-3.44538	0.19691
H	2.19702	-1.4393	2.19318	C	3.89847	-2.43315	1.32197
C	3.20812	1.50154	1.15427	C	7.33698	0.88826	-1.24399
H	2.71415	1.77508	2.08255	H	8.39209	0.99233	-0.98765
C	4.59607	1.51162	1.09662	H	7.09129	1.57918	-2.06042
H	5.19312	1.78065	1.96219	H	7.14081	-0.14187	-1.56653
H	4.45545	-2.44847	2.25449	C	2.45336	-2.38585	-1.05824
C	-1.31407	0.2989	0.05318	H	1.88386	-2.38227	-1.98373
C	-1.90037	1.58663	0.08784	C	3.72321	-2.96395	-1.0264
C	0.93083	1.20813	0.10458	H	4.14022	-3.39798	-1.93115
C	-1.08046	2.7171	0.17153	C	4.49422	0.81216	-1.21845
H	-4.29233	3.35759	-0.00989	H	4.9709	0.53639	-2.15151
C	-5.98419	0.42826	-0.22373	O	-7.30019	0.06742	-0.34126
C	-4.43689	2.28534	-0.04134	C	-4.92134	-0.48512	-0.18175
C	-5.74007	1.80667	-0.15202	H	-5.12543	-1.54155	-0.26374
H	-6.58399	2.48713	-0.19344	C	-7.62858	-1.3127	-0.42011
C	-0.52235	-2.35808	0.08127	H	-8.71552	-1.35522	-0.50138
C	-1.94416	-2.05295	0.03676	H	-7.3111	-1.853	0.48108
C	1.8863	-1.82353	0.09723	H	-7.18069	-1.78263	-1.30518

Table S5. Minimum energy structure of *DimethoxyASI* Dimer 1 (nuclear repulsion energy: 13441.0823277367 Hartrees)

C	7.373681	-0.768140	-0.999434	C	-8.508696	2.249555	0.560790
C	5.350653	-1.604670	-2.039854	H	-9.460044	2.755382	0.390125
C	6.707440	-1.741696	-1.756706	H	-8.354793	2.123717	1.640423
H	7.271894	-2.593218	-2.122115	H	-7.694759	2.855609	0.143017
C	6.682967	0.352742	-0.518314	O	3.781237	-5.151569	1.166085
O	8.706071	-0.998617	-0.783354	H	-8.659167	-1.251647	-1.257248
H	4.879238	-2.375199	-2.636301	O	-8.614316	0.995079	-0.098498
C	4.634767	-0.496068	-1.569337	H	-3.722222	3.110179	4.003900
C	5.316787	0.491648	-0.797893	H	3.774325	-1.525924	3.720009
H	7.216879	1.098821	0.049197	H	-6.858798	-2.921607	-1.348204
C	9.455997	-0.047009	-0.040273	C	-7.719143	-1.072896	-0.745997
C	3.235910	-0.065269	-1.719056	C	-7.549029	0.135291	-0.055949
C	4.341191	1.544459	-0.454061	H	-6.221452	1.354903	1.115235
H	9.071537	0.057270	0.982544	H	-4.939592	1.703154	2.426026
H	9.458950	0.934122	-0.532101	C	-3.228777	2.258197	3.544335
H	10.474782	-0.434893	-0.004016	C	2.762416	-1.475128	3.328743
C	2.141322	-0.584292	-2.419220	H	3.249703	-0.035792	1.804258
C	3.110956	1.169512	-1.039228	H	1.948716	-2.908412	4.724739
C	4.426225	2.719176	0.302243	H	-1.350638	2.568500	4.603160
C	2.187459	-1.794717	-3.184653	H	-4.825054	-6.496034	-2.302454
C	0.899081	0.175353	-2.383037	C	-6.692197	-2.013144	-0.783793
C	1.915524	1.938631	-0.971397	C	-6.347945	0.407444	0.613829
C	3.219372	3.521928	0.424507	C	-3.907913	1.471860	2.652026
C	5.613961	3.154613	0.975366	H	0.960629	0.113809	0.914773
C	1.096380	-2.245752	-3.878365	C	2.464397	-0.638314	2.251885
H	3.106937	-2.363041	-3.221305	H	-0.341250	-2.756538	3.815865
C	-0.210791	-0.343311	-3.139976	C	1.744286	-2.245623	3.888845
C	0.782229	1.396995	-1.669515	H	-0.241090	0.637718	3.620489
C	2.001796	3.153697	-0.213742	C	-1.882123	1.948982	3.886643
C	3.296927	4.702014	1.247025	H	-2.418471	-7.115035	-1.991068
H	6.513215	2.560047	0.903043	C	-4.164076	-5.834436	-1.749711
C	5.634496	4.296119	1.731906	H	-5.678776	-4.393469	-1.398077
C	-0.120936	-1.507075	-3.855760	C	-5.482403	-1.768226	-0.122132
H	1.158429	-3.166866	-4.450542	C	-5.320537	-0.544441	0.591611
H	-1.140051	0.211187	-3.147130	C	-3.298933	0.324207	2.047410
C	-0.515899	2.108349	-1.729853	C	1.172367	-0.551546	1.744491
C	0.861894	4.092502	0.004372	C	0.451558	-2.162795	3.369321
H	2.409009	5.303905	1.384343	C	-1.258634	0.863445	3.330318
C	4.455068	5.078931	1.873092	C	-2.797550	-6.191720	-1.562685
H	6.548077	4.597744	2.236418	H	-0.922976	-5.660831	-0.726018
H	-0.980183	-1.866827	-4.413635	C	-4.640434	-4.654487	-1.243846
C	-0.643829	3.241487	-2.522371	C	-4.221735	-2.520835	-0.018201
C	-1.648740	1.649632	-1.045695	C	-3.962274	-0.537739	1.167373
C	0.821504	5.329615	-0.651622	C	0.147421	-1.325565	2.298055
C	-0.104157	3.817649	0.974150	C	-1.924607	-0.004274	2.393106
H	4.470369	5.973132	2.489106	C	-3.796540	-3.759419	-0.508708
C	-1.853949	3.926979	-2.658586	C	-3.329127	-1.726323	0.739058
H	0.220827	3.602511	-3.071678	C	-1.268682	-1.151875	1.865938
H	-1.589724	0.762542	-0.424649	C	-1.959774	-5.376129	-0.849985
C	-2.847453	2.332943	-1.144861	C	-2.412511	-4.141393	-0.262982
H	1.581214	5.555179	-1.395745	C	-1.985177	-2.081478	1.041461
C	-0.151493	6.281494	-0.351121	H	0.674317	-2.345278	-0.698324
C	-1.083562	4.760423	1.264556	H	-0.615434	-5.392228	2.023490
H	-0.090394	2.872305	1.505792	C	-1.527923	-3.328369	0.492399
C	-2.968193	3.472095	-1.951075	C	-0.134610	-3.799359	0.658008
H	-1.900801	4.803538	-3.293386	H	2.992785	-3.067650	-0.423976
H	-3.724892	1.985414	-0.609966	C	0.180400	-4.888836	1.480799
C	-1.114902	5.997262	0.617556	H	1.731711	-6.171188	2.272154
H	-0.152222	7.236639	-0.869073	C	0.895109	-3.177933	-0.041007
H	-1.833875	4.526374	2.014356	C	2.210702	-3.593785	0.109636
O	-4.203468	4.061049	-1.975656	C	1.487793	-5.333077	1.627278
H	-1.881511	6.725373	0.866522	C	4.869608	-4.534348	0.487451
C	-4.394266	5.188836	-2.820032	H	5.764189	-5.064272	0.817368
H	-5.439552	5.477573	-2.701234	H	4.767780	-4.634406	-0.600284
H	-4.204666	4.937279	-3.871312	H	4.957083	-3.472945	0.744703
H	-3.749961	6.026162	-2.523827	C	2.519223	-4.673884	0.945641

Table S6. Minimum energy structure of *DimethoxyASI* Dimer 2 (nuclear repulsion energy: 12461.4161798055 Hartrees)

O	1.545646	-3.988804	-2.018531	H	1.929366	-4.081191	2.117432
C	-0.290851	-2.438760	-1.561742	C	7.549450	-3.866450	0.323881
H	0.284304	-1.600460	-1.921992	H	7.641224	-3.791555	2.481729
C	2.340223	-2.920316	-2.518110	H	-3.287780	3.371103	2.815385
H	3.286164	-3.372174	-2.819143	H	-2.529818	2.166059	1.741316
H	2.529302	-2.166901	-1.744405	H	-1.871760	2.446356	3.387218
H	1.869896	-2.447069	-3.389780	H	0.011233	-2.523078	2.519376
C	0.283096	-3.717970	-1.568033	O	11.063251	-1.227901	-0.617184
C	-1.600720	-2.275749	-1.094787	C	6.054390	2.785770	-0.325955
C	-0.441094	-4.824053	-1.102252	C	2.343285	3.399848	0.627442
C	-2.343407	-3.399911	-0.627700	C	4.882706	0.722253	0.381226
C	-2.480191	-1.090213	-1.011841	C	1.600582	2.275652	1.094448
C	-1.743815	-4.665672	-0.637189	C	7.371489	0.683304	-0.177395
H	0.030688	-5.800847	-1.123091	C	9.865519	-0.607560	-0.391838
C	-3.691206	-2.933001	-0.270740	H	5.656123	-1.793191	-1.610739
C	-3.732039	-1.543621	-0.539311	C	7.947123	0.450955	-1.433123
C	-2.306061	0.256782	-1.349651	H	7.430077	0.769372	-2.333641
H	-2.273441	-5.549744	-0.306534	C	9.164952	-0.194931	-1.533223
C	-4.839628	-3.579501	0.196982	H	9.612010	-0.390654	-2.503696
C	-4.882685	-0.722257	-0.381235	H	7.244126	-3.673639	-1.795831
C	-3.443442	1.144509	-1.158528	H	8.235151	-4.703130	0.227616
C	-1.082556	0.803856	-1.857325	C	3.732050	1.543621	0.539321
C	-4.884491	-4.972487	0.531053	C	3.691202	2.933004	0.270753
C	-6.054386	-2.785785	0.325948	C	6.078349	1.394132	-0.047281
C	-6.078349	-1.394138	0.047293	C	4.839638	3.579494	-0.196986
C	-4.699316	0.667024	-0.688730	C	11.829148	-1.645243	0.506209
C	-3.236204	2.542180	-1.443886	C	7.252905	3.483974	-0.714321
H	-0.231573	0.155955	-2.005281	C	1.743481	4.665514	0.636582
C	-0.954257	2.135583	-2.145968	C	4.699206	-0.667084	0.688685
C	-6.046463	-5.576452	0.930626	C	0.290462	2.438494	1.560748
H	-3.976882	-5.557969	0.471386	C	2.480193	1.090188	1.011811
C	-7.252902	-3.484026	0.714265	C	8.067799	0.259100	0.950533
C	-7.375029	-0.692269	0.172708	C	9.312510	-0.370281	0.867884
C	-5.759031	1.704961	-0.542431	C	4.884517	4.972472	-0.531086
H	-4.041228	3.241285	-1.264560	H	11.288297	-2.387070	1.106786
C	-2.046274	3.019313	-1.923734	H	12.733037	-2.099355	0.098122
H	-0.011341	2.522984	-2.519781	H	12.104677	-0.793146	1.140492
C	-7.252475	-4.821720	1.006837	H	8.182392	2.932352	-0.769615
H	-6.053311	-6.633605	1.180312	C	7.252491	4.821662	-1.006921
H	-8.182403	-2.932429	0.769549	C	0.440509	4.823731	1.101040
C	-8.065343	-0.280068	-0.963792	H	2.273090	5.549647	0.306042
C	-7.954117	-0.454725	1.426076	C	3.443342	-1.144554	1.158212
C	-6.323219	2.282935	-1.685083	C	5.757307	-1.706041	0.538704
C	-6.110981	2.220951	0.708007	H	-0.284726	1.600144	1.920818
H	-1.929641	4.081036	-2.118345	C	-0.283774	3.717584	1.566554
H	-8.177673	-5.312269	1.295058	C	2.306014	-0.256825	1.349471
C	-9.272913	0.398361	-0.856990	H	7.640327	0.439936	1.932331
H	-7.645544	-0.469168	-1.946712	C	2.046076	-3.019436	1.923042
H	-7.433455	-0.782759	2.321678	H	4.040982	-3.241417	1.263714
C	-9.173950	0.198196	1.550662	C	6.986891	-3.284922	-0.813849
H	-6.052500	1.907097	-2.667423	C	7.218346	-3.356995	1.580112
C	-7.213730	3.339425	-1.562882	H	6.063423	-1.897393	2.654523
C	-7.002608	3.287344	0.831768	C	-2.341478	2.919488	2.515160
H	-5.672754	1.783111	1.600408	C	0.954140	-2.135669	2.145549
C	-9.839674	0.640047	0.399823	H	0.231578	-0.155955	2.005264
H	-9.774075	0.727028	-1.760138	H	9.816243	-0.675964	1.776929
H	-9.618566	0.386202	2.522666	H	3.976919	5.557971	-0.471411
C	-7.558179	3.857643	-0.312478	C	6.046491	5.576412	-0.930695
H	-7.642294	3.780004	-2.460128	H	6.053348	6.633559	-1.180403
H	-7.257056	3.667323	1.816960	H	8.177690	5.312192	-1.295175
O	-11.022851	1.295114	0.605371	H	-0.031430	5.800459	1.121534
H	-8.249363	4.691706	-0.235066	C	3.236024	-2.542290	1.443262
C	-11.763517	1.734518	-0.526461	C	6.093958	-2.222483	-0.714841
H	-12.660574	2.210176	-0.127843	C	6.332651	-2.284688	1.675415
H	-12.052752	0.891441	-1.166649	O	-1.546647	3.988179	2.016237
H	-11.197331	2.464374	-1.118110	C	1.082503	-0.803898	1.857138
